# CD44‐associated radioresistance of glioblastoma in irradiated brain areas with optimal tumor coverage

**DOI:** 10.1002/cam4.2714

**Published:** 2019-11-19

**Authors:** Wei‐Hsiu Liu, Jang‐Chun Lin, Yu‐Ching Chou, Ming‐Hsien Li, Jo‐Ting Tsai

**Affiliations:** ^1^ Department of Neurological Surgery Tri‐Service General Hospital and National Defense Medical Center Taipei Taiwan ROC; ^2^ Department of Surgery School of Medicine National Defense Medical Center Taipei Taiwan ROC; ^3^ Graduate Institute of Clinical Medicine College of Medicine Taipei Medical University Taipei Taiwan ROC; ^4^ Department of Radiation Oncology Shuang Ho Hospital Taipei Medical University Taipei City Taiwan ROC; ^5^ Department of Radiology School of Medicine College of Medicine Taipei Medical University Taipei Taiwan ROC; ^6^ School of Public Health National Defense Medical Center Taipei Taiwan ROC

**Keywords:** cancer stem‐like cells, glioblastoma, planning targeted volume, radiotherapy

## Abstract

Glioblastoma multiforme (GBM) requires radiotherapy (RT) as its definitive management. However, GBM still has a high local recurrence rate even after RT. Cancer stem‐like cells (CSCs) might enable GBM to evade irradiation damage and cause therapeutic failure. The optimal RT plan should achieve a planning target volume (PTV) coverage of more than 95% but cannot always meet the requirements. Here, we demonstrate that irradiation with different tumor coverage rates to different brain areas has similar effects on GBM. To retrospectively analyze the relationship between PTV coverage and the survival rate in 26 malignant glioblastoma patients, we established primary cell lines from patient‐derived malignant glioblastoma cells with the PTV_95_ (PTV coverage of more than 95%) program (GBM‐MG1 cells) and the Non‐PTV_95_ (poor PTV coverage of less than 95%) program (GBM‐MG2 cells). The clinical results of PTV_95_ and Non‐PTV_95_ showed no difference in the overall survival (OS) rate (*P *= .390) between the two different levels of PTV coverage. GBM‐MG1 (PTV_95_ program) cells exhibited higher radioresistance than GBM‐MG2 (Non‐PTV_95_ program) cells. CD44 promotes radioresistance, CSC properties, angiogenesis and cell proliferation in GBM‐MG1 (PTV_95_ program) cells. GBM patients receiving RT with the PTV_95_ program exhibited higher radioresistance, CSC properties, angiogenesis and cell proliferation than GBM patients receiving RT with the Non‐PTV_95_ program. Moreover, CD44 plays a crucial role in these properties of GBM patients with the PTV_95_ program.

## Graphical abstract

Glioblastoma multiforme (GBM) patients receiving radiotherapy (RT) with the PTV95 program exhibited higher radioresistance, CSC properties, angiogenesis and cell proliferation than GBM patients receiving RT with the Non‐PTV95 program. Moreover, CD44 plays a crucial role in these properties of GBM patients with the PTV95 program.

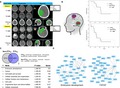

## 1 INTRODUCTION

Malignant glioma is the most common primary malignant central nervous system (CNS) tumor in adults. Glioblastoma multiforme (GBM) is the World Health Organization (WHO) grade IV malignant glioma.^1^ It is the most devastating brain cancer due to its resistance to all current therapy, including operation, radiotherapy (RT), chemotherapy, and immunotherapy.^2^ RT is highly effective, destroying cancer cells that may exist around the surgical tumor bed. In clinic, the standard therapy for GBM is complete surgical resection followed by concurrent chemoradiotherapy and adjuvant chemotherapy for six months.^3^ However, even after standard clinical treatment, 5‐year overall survival of GBM is less than 10%.^4^ One of the major reasons for therapeutic failure is that cancer stem‐like cells (CSCs) are present in the central nervous system (CNS), which might enable glioblastoma multiforme (GBM) to escape from irradiation‐induced damage.^5^ Irradiated GBM cells have been thought to act as CSCs, with high self‐renewal capacity, relative quiescence, and protection by the niche, thus underlying tumor recurrence and radioresistance.^6^, ^7^ Human irradiated glioblastoma specimens were found to be enriched in CSCs.^8^ Moreover, fractionated ionizing radiation (IR), which is similar in use to clinical RT, enhanced the portion of the CSC population in vivo.^9^ However, it is still not understood whether the enhancement of such mechanisms is inherent in the adaption of CSCs to repeated radiation.

CSCs maintain tumor growth through self‐renewal ability and generate a bulky tumor with cooperation from different locations of the brain. RT technology improves each passing day to optimize the irradiated tumor coverage. In standard RT dosimetry, planning target volume (PTV) coverage should be at least 95%; however, this goal cannot always be met due to the need to spare the adjacent organs at risk (OARs). Therefore, irradiated brain areas around the OAR might have poor PTV coverage. However, different tumor coverage rates to different irradiated regions of the have similar effects on GBM. Malignant gliomas frequently exhibit transiently complete remission by conventional imaging; however, resistant glioma cells can be undetectable by such imaging technique. These cells present the ability to regrow the primary tumor and thereby promote recurrent disease.^10^ Thus, further identifying which markers affect GBM at different regions of the brain to induce different levels of CSC properties is another method that can be used to treat GBM.

CD44 is a cell surface adhesion receptor that regulates the progression and metastasis of cancer cells via the recruitment of CD44 to the cell surface and is highly expressed in many cancers.^11^ A previous study reported that the expression of CD44 correlated with the tumor subtype and serves as a marker of CSCs.^11^ For example, it has been reported that CD44‐variant CSCs induce chemoresistance and enhance tumorigenicity in colorectal cancer cells.^12^ Glioblastoma CSCs differentiate not only into neural lineages but also into mesenchymal stem cells (MSCs).^13^ CD44 is an important cell surface marker that is expressed on MSCs.^14^ CD44 is also one of the nine markers that can be subjected to multicolor flow cytometry analysis of the gliomasphere‐ an established model of glioblastoma stem‐like cells.^15^ Moreover, glioblastoma CSCs with high levels of CD44 expression promotes not only tumor invasion but also rapid tumor progression and short survival in patients with GBM.^16^

In our study, we first identified clinical patients with glioblastoma CSCs based on our clinical observation. The results have increasingly suggested that GBM contains CSCs, which are radioresistant and result in therapeutic failure. We hypothesized that CD44 induces the radioresistance of GBM due to the increased existence of CSCs in better tumor coverage of the irradiated brain region.

## 2 METHODS

### 2.1 Patient characteristics and targeted volume definition

Patients with glioblastoma were treated for primary brain tumors and perifocal edema using methods approved by the multidisciplinary CNS tumor board at Shuang Ho Hospital. The inclusion criteria included the following: pathology‐proven primary brain high‐grade glioma according to the WHO Classification of Tumors of the Central Nervous System^1^; an Eastern Cooperative Oncology Group (ECOG) performance score of 0, 1, 2 or 3; and age 20 to 90 years. All procedures of patient acquisition were approved by the Institutional Review Committee at Shuang Ho Hospital, Taipei Medical University. We evaluated tumor response according to the Response Evaluation Criteria In Solid Tumors (RECIST).^17^ The characteristics of these patients were summarized in Table 1.

**Table 1:** Patients and tumor characteristics (N = 26)

Patient characteristic	Non‐PTV_95_ (N = 15)	PTV_95_ (N = 11)	*P* value^a^
	N (%)		
Age, M ± SD	58.53 ± 14.71	57.45 ± 12.11	.844^a^
Sex			.951^a^
Female	7 (46.7)	5 (45.5)	
Male	8 (53.3)	6 (54.5)	
ECOG			.479^b^
0	3 (20.0)	1 (9.1)	
1	11 (73.3)	7 (63.6)	
2	1 (6.7)	3 (27.3)	
Surgery type			.315^b^
Gross total resection	11 (73.3)	6 (54.5)	
Subtotal resection	0 (0)	2 (18.2)	
Biopsy only	4 (26.7)	3 (27.3)	
Tumor side of brain			.683^b^
Right side	9 (60.0)	8 (72.7)	
Left side	6 (40.0)	3 (27.3)	
Chemotherapy			.356^b^
None	4 (26.7)	1 (9.1)	
Temozolomide	11 (73.3)	10 (90.9)	

Abbreviation: M ± SD: Mean ± deviation.

a Independent *t* test or chi‐square test.

b Fisher's exact test; ECOG, Eastern Cooperative Oncology Group performance score.

### 2.2 Sphere‐formation and self‐renewal assays

Sphere formation and self‐renewal assays were performed essentially as previously described.^18^ See the Supplementary material.

### 2.3 Quantitative real‐time reverse‐transcriptase (qPCR)

qPCR was performed according to previously described methods.^18^ Table S1 shows the sequences of primers used for real‐time PCR experiments. See the Supplementary material.

### 2.4 Western blot assays

Western blot assays were performed according to previously described methods.^18^ The primary antibodies that were used are listed in Table S2. See the Supplementary material.

### 2.5 Immunohistochemistry staining

Immunohistochemistry staining assays were performed according to previously described methods.^18^ The primary antibodies that were used are listed in Table S2. See the Supplementary material.

### 2.6 Annexin V apoptosis staining

Annexin V apoptosis staining was performed essentially as previously described.^18^ See the Supplementary material.

### 2.7 Irradiation and clonogenic assay

Briefly, cells in the control group and post‐IR group were administered with irradiation 5 Gy. The clonogenic assay was performed according to previously described methods.^22^

### 2.8 Microarray, IPA and PCA analysis

Affymetrix U133 plus 2.0 Microarray analysis was performed as described.^18^ PTV_95_ and Non‐PTV_95_ microarray were obtained from Sturm D, et al.^19^ Differentially expressed mRNAs were identified by using the t‐test procedure within significance analysis of microarrays. We classified these GBM samples found at frontal lobe, frontal/temporal, hemispheric, parietal lobe, parieto‐occipital, or temporo‐parietal region as PTV_95_ group (12 patients). We also classified GBM samples found at pons, thalamic, ventricular, temporal lobe, cerebellar, or central region as Non‐PTV_95_ group (12 patients). The Venn diagram, PCA and heatmap analysis were performed with software Orange (https://orange.biolab.si). The differential expressed genes were analyzed with software Ingenuity Pathways Analysis (QIAGEN Inc, https://www.qiagenbio-informatics.com/products/ingenuity-pathway-analysis ). Twenty‐four samples obtained from NCBI GEO database, including 12 PTV samples and 12 Non‐PTV samples were applied to PCA analysis. For demonstrating that GBM MG1 or MG2 cell lines are similar to PTV or Non‐PTV respectively, we further included our GBM‐MG1 and GBM‐MG2 samples and compared with clinical samples.

### 2.9 Statistical analyses

The clinical data of patients were collected retrospectively from medical records, and a total of 26 patients were included in this analysis. Statistical analysis was performed using the Statistical Package for Social Sciences 20 (SPSS Inc). Overall survival was the primary endpoint. The total mortality and progression‐free survival (PFS) rates were calculated from the first day of RT by the Kaplan–Meier method. Univariate and multivariate Cox regression analyses were done for PFS in GBM patients. Cox proportional hazards model analysis was used to evaluate the differences between PTV coverage. A *P* < .05 was considered significant for both clinical and laboratory studies.

## 3 RESULTS

### 3.1 GBM in different brain areas exhibits different gene expression profiles

**Figure 1:**
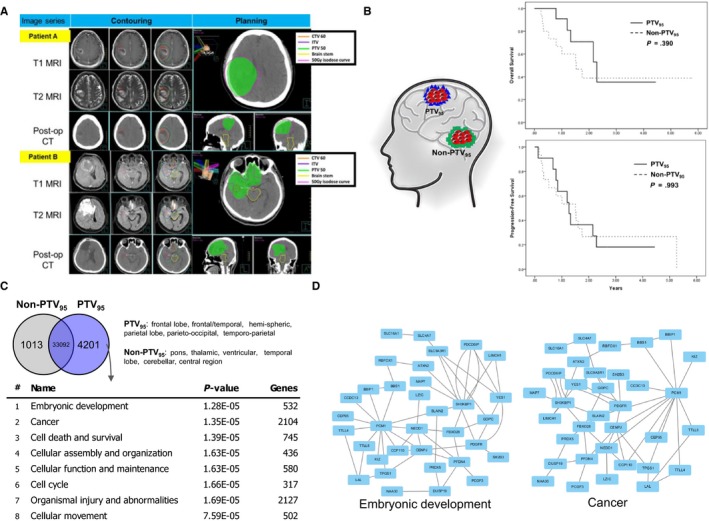
PTV_95_ GBM shows more stem cell and cancer properties in transcription profile. A, MRI image analysis of PTV_95_ and non‐PTV_95_ GBM patient. B, (Left): Study design showing that PTV_95_ and non‐PTV_95_ patients show similar outcome under different radiation dosage. (Right): Overall survival and progression‐free survival analysis of PTV_95_ and non‐PTV_95_ GBM patients. C, Transcriptional comparison of tissues sampling from PTV_95_ and non‐PTV_95_ region. (Upper) upregulated gene in Non‐PTV_95_ is shown in gray, meanwhile upregulated genes in PTV_95_ is shown in blue. Non‐PTV_95_ tissues have 1013 unique upregulated gene, and PTV tissues have 4201 unique upregulated genes. (Lower) IPA analysis of PTV_95_‐specific upregulated genes. Biological classification shows top five significant functions and shows significance and the total number in each function. D, Transcriptional network of top two functions, which explaining PTV_95_ may associate with stem cell and tumor malignancy

First, we collected data from 26 patients with glioblastoma who received RT and regular follow‐up brain magnetic resonance imaging (MRI) every 3 months. Eleven patients had achieved PTV coverage of more than 95% (PTV_95_), while 15 patients had poor PTV (Non‐PTV_95_) coverage of less than 95%. PTV coverage of at least 95% should be achieved according to standard RT planning, as in patient A (Figure 1A); however, sometimes this requirement cannot be met due to the need to spare the adjacent OARs, as in patient B (Figure 1A). From these examples, we could clearly explain the indication of RT planning with poor coverage due to preventing damage to the brain stem or optic chiasms in patient B.

There was no statistically significant difference between the two groups regarding patient characteristics, as shown in Table 1. Therefore, we hypothesize that glioblastomas in PTV_95_ are different from those in Non‐PTV_95_ and that these different factors could further educate CSCs to induce radioresistance, resulting in a similar rate of tumor recurrence and disease prognosis. (Figure 1B, left). Thus, the 3‐year overall survival (OS) rates of the PTV_95_ and Non‐PTV_95_ groups were 36.9% and 38.6%, respectively, from the start date of RT (Figure 1B, right upper panel, *P* = .390). Additionally, patients with PTV_95_ had a 3‐year progression‐free survival (PFS) rate of 18.9%, which was similar to the rate of 21.8% observed in the Non‐PTV_95_ group (Figure 1B, right lower panel, *P* = .993). However, there were no differences in OS or PFS between the 2 different levels of PTV coverage of RT. Even after adjusting for patient characteristics or RT planning factors, including age, sex, surgery type, and tumor side of the brain, the level of PTV coverage had no effect on OS or PFS risk, as shown in Table 2. Regardless of whether the PTV coverage reaches 95%, it has no effect on the patients’ clinical outcomes, and this finding is in contrast to the widely accepted importance of PTV coverage. The lack of an effect might be related to CSC activation after RT. However, there are no differences between malignant glioblastomas with PTV_95_ and Non‐PTV_95_.

**Table 2:** Cox proportional hazards model analysis for the association between PTV coverage (PTV_95_ vs Non‐PTV_95_) with overall survival and progression risk of glioblastoma

Risk factor	Overall survival risk		Progression risk	
	HR (95% CI)	*P* value	HR (95% CI)	*P* value
PTV_95_ vs Non‐PTV_95_	0.53 (0.17‐1.63)	.266	0.81 (0.33‐1.98)	.637
Model 2	0.38 (0.11‐1.36)	.136	0.70 (0.28‐1.79)	.458
Model 3	0.33 (0.08‐1.30)	.113	0.44 (0.16‐1.24)	.120

Abbreviations: CI, confidence interval; HR, Hazard ratio; ref, reference group.

Model 1: crude HR(95% CI).

Model 2: PTV_95_ vs Non‐PTV_95_ adjustment for age and sex.

Model 3: PTV_95_ vs Non‐PTV_95_ adjustment for age, sex, surgery type, and tumor side of brain.

To identify the transcriptional difference, we collected microarray data from 24 GBM patients, including 12 PTV_95_ and 12 Non‐PTV_95_ patients from Sturm et al,^19^ and we compared the uniquely expressed genes in each group and selected the highly expressed genes in PTV_95_ for further study. We used the knowledge‐based software ingenuity pathway analysis (IPA) to predict biological function. The IPA showed that the highly expressed genes are associated with embryonic development, cancer, cell death and survival, cellular assembly and organization, cellular function and maintenance, cell cycle, organismal injury and abnormalities, and cellular movement (Figure 1C); within the classification, cancer and organismal injury and abnormalities were most likely observed in cancer cells, which suggests that PTV_95_ tumors possess typical cancer gene signatures. GBM at the frontal lobe, frontal‐temporal area, hemisphere, parietal lobe, parieto‐occipital area, and temporo‐parietal region is defined as PTV_95_. Non‐PTV_95_ includes tumors at the pons, thalamus, ventricular, temporal lobe, cerebellar and central region. Remarkably, the cells show a highly significant correlation with embryonic development, which indicates that a PTV_95_ tumor exhibits some stem cell properties. Stem cell properties are usually associated with malignant cancer properties,^20^ implying that PTV_95_ may associate with malignant cancer properties, such as high proliferation, drug resistance, or radiation resistance. Furthermore, IPA revealed the transcriptional network to provide greater detail of the central regulatory genes involved in embryonic development and cancer categories (Figure 1D). Therefore, even though the outcomes between PTV_95_ and Non‐PTV_95_ are difficult to distinguish, we discovered the transcriptional signature in PTV_95_ and identified PTV_95_ tumors as having more CSCs than Non‐PTV_95_ tumors.

### 3.2 GBM‐MG1 (PTV_95_ program) cells present higher radioresistance, cancer stem‐like properties, angiogenesis and cell proliferation than GBM‐MG2 (Non‐PTV_95_ program) cells

**Figure 2:**
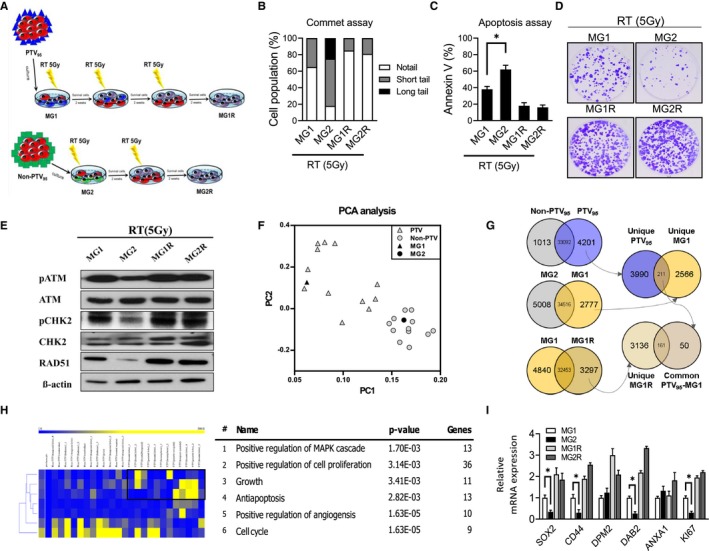
The PTV_95_ GBM tissues show similar properties with radio‐resistant cells. A, Scheme showing in vitro model MG1 and MG2 for mimic PTV_95_ and non‐PTV_95_ GBM, respectively. To investigate the effect of radiation in the cell model, we establish radiation‐resistant MG1R and MG2R. B, Comet assay and (C) Annexin V assay were used to compare the radioresistance among MG1, MG2, MG1R, and MG2R GBM cells after radiation (5 Gy). D, Colony formation assays were performed to compare the radioresistance among MG1, MG2, MG1R, and MG2R GBM cells after radiation (5 Gy). E, Western blotting shows the level of DNA repair protein among MG1, MG2, MG1R, and MG2R GBM cells after radiation (5 Gy). F, PCA analysis demonstrated that the transcriptional profile of GBM‐MG1 or GBM‐MG2 is similar to that of PTV or Non‐PTV patients. G, Venn diagram displaying overlap of significant genes found in the Affymetrix U133 plus 2.0 microarray experiment. PTV_95_ and non‐PTV_95_ clinical data were obtained from Sturm et al^19^ GBM cell lines MG1, MG2, and MG1R cells were also analyzed with Microarray. Genes expressed with fold‐change >1.5 in the microarray experiment were counted. H, The overlap between unique MG1R and common PTV_95_‐MG1 were selected with heatmap clustering and then applied into Ingenuity Pathway Analysis. The classification shows six significant biological functions, significance and gene number of each function. I, Quantitative PCR showing the expression of selected upregulated genes, including SOX2, CD44, DPM2, DAB2, ANXA1, and KI67. All data are presented as the mean ± SD, n = 10, *P < .05 by Student's *t* test

To verify the radiation effect, we established GBM primary cell lines (MG1) from one patient with GBM receiving the PTV_95_ program. IR was used to treat the primary MG1 cells, and the residual cells were cultured. The irradiated cells were again subjected to the same treatments twice, and the corresponding age‐ and passage‐matched irradiated cell lines were established (MG1R). Moreover, we also established another GBM primary cell line (MG2) from one patient with GBM receiving the Non‐PTV_95_ program. The MG2 cell line also received IR, and the residual cells were cultured. The irradiated cells were again subjected to the same treatments one time, and the corresponding age‐ and passage‐matched irradiated cell lines were established (MG2R) (Figure 2A). Furthermore, in comet assays performed 24 hours after irradiation, GBM‐MG1 cells showed modest double‐strand break (DSB) accumulation, while GBM‐MG2 cells exhibited severe DNA damage (Figure 2B). In addition, GBM‐MG1R and GBM‐MG2R cells had the same level of radioresistance with less DNA damage (Figure 2B). Annexin V staining revealed that GBM‐MG1 cells survived significantly more than GBM‐MG2, and GBM‐MG1R cells and that GBM‐MG2R cells had a similar level of less apoptosis after IR (5 Gy) (Figure 2C). Consistently, in radiobiological clonogenic assays, the survival abilities of GBM‐MG1 cells were significantly more than GBM‐MG2, GBM‐MG1R cells and that GBM‐MG2R cells had a similar level of higher radioresistance after IR (5 Gy) (Figure 2D). Moreover, GBM‐MG1 cells with high radioresistance exhibited constitutive ATM, CHK2 kinase phosphorylation and RAD51; moreover, GBM‐MG2 cells with lower radioresistance exhibited lower ATM and CHK2 kinase phosphorylation levels. ATM and CHK2 kinase phosphorylation were significantly increased in GBM‐MG1R and GBM‐MG1R cells; however, GBM‐MG1R and GBM‐MG1R cells had similar levels of ATM, CHK2 kinase phosphorylation and RAD51 (Figure 2E). Collectively, these data suggest that the positive selection of GBM‐MG1R and GBM‐MG2R cells by IR relies on intrinsic radioresistance and the increased hyperactivation of DNA damage response (DDR) effectors after irradiation.

To further discover the underlying mechanism and to address why PTV_95_ shows higher radioresistance, we collected the microarray data of 24 GBM patients from Sturm et al^19^ and classified these GBM samples into the PTV_95_ (12 patients) or Non‐PTV_95_ (12 patients) group. The PTV_95_ group includes the GBM location at the frontal lobe, frontal/temporal, hemispheric, parietal lobe, parieto‐occipital, and temporoparietal regions. Moreover, the Non‐PTV95 group includes the pons, thalamic, ventricular, temporal lobe, cerebellar, and central regions. To examine the transcriptional difference among PTV_95_, Non‐PTV_95_, GBM‐MG1 and GBM‐MG2 cell lines, we performed the PCA analysis by using Affymetrix microarray data. PCA analysis revealed that dot of PTV_95_ and Non‐PTV_95_ samples forms individual cluster (Figure 2F), indicating that PTV_95_ or Non‐PTV_95_ samples shows its uniqle transcription pattern. Meanwhile, we observed dot of GBM‐MG1 cell was surrounded by the cluster of PTV, indicating that GBM‐MG1 cells are similar with PTV_95_ samples. In addition, PCA analysis also showed GBM‐MG2 cells are also similar with Non‐PTV_95_ Samples (Figure 2F). Then, we used a Venn diagram to obtain the 4201 upregulated genes in PTV_95_ (not in Non‐PTV95) (Figure 2G, left upper). Then, we obtained 2777 upregulated genes in the PTV_95_‐derived cell line GBM**‐**MG1 compared to the Non‐PTV_95_‐derived cell line GBM**‐**MG2 (Figure 2G, left middle). To further filter the influence of patients, we used 2777 upregulated genes in GBM**‐**MG1 cells to refine our candidates among the 4201 genes. A total of 211 common genes from the overlap of unique PTV_95_ and unique GBM**‐**MG1 cells were suggested to play important roles in the PTV_95_ region (Figure 2G, right upper). Since cancer stem cells are reported as radioresistant cells and PTV_95_ shows some stem cell properties from the IPA (Figure 1C), we further used 3297 upregulated genes in the radioresistant cell line GBM**‐**MG1R (Unique MG1R) to refine the common PTV_95_‐MG1 overlap (Figure 2G, left lower). In the final Venn diagram, we obtained 161 candidates from the sequent analysis (Figure 2G, right lower). Heatmap and clustering analysis revealed that three clusters from 161 candidates are enriched in the PTV patients (Figure 2H, left). We further analyzed candidate genes of these three cluster with IPA analysis and identified their biological functions as follows: positive regulation of the MAPK cascade, positive regulation of cell proliferation, growth, anti‐apoptosis, positive regulation of angiogenesis, and cell cycle (Figure 2H, right). IPA also showed the transcriptional network of the first two categories: positive regulation of the MAPK cascade and positive regulation of cell proliferation (Figure 2H). To confirm the central regulator within the identified gene network, we performed qPCR to detect mRNA expression. qPCR analysis showed that GBM‐MG1 cells had higher expression of SOX2, CD44, DPM2, DAB2, ANXA1, and Ki67 than GBM‐MG2 cells (Figure 2I).

### 3.3 CD44 promotes cancer stem‐like properties and enhances the radioresistance, angiogenesis, and proliferation of GBM‐MG1 (PTV_95_ program) cells

**Figure 3:**
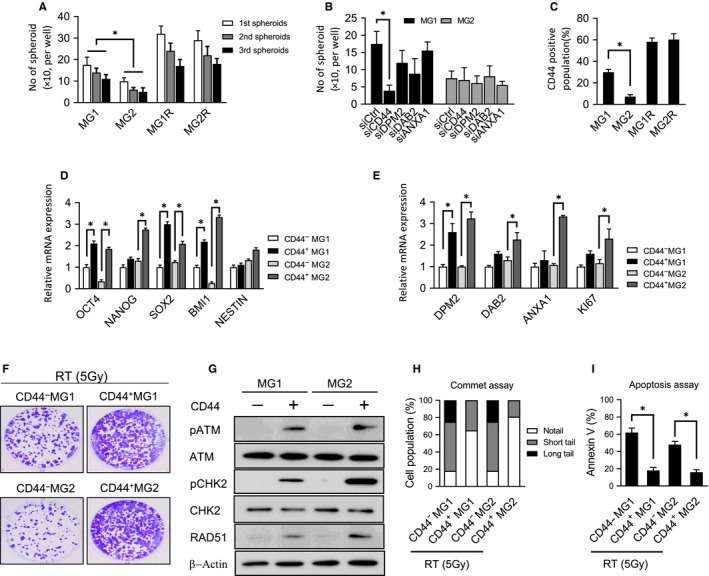
CD44 plays a crucial role in PTV_95_ GBM properties. A, Spheroid formation assay showing GBM‐MG1 has higher spheroid formation ability than GBM‐MG2. Similarly, both GBM‐MG1R and GBM‐MG2R show higher tendency in spheroid formation. B, Spheroid formation assay of GBM‐MG1 and GBM‐MG2 cells showing the effect of siCD44, siDPM2, siDAB2, siANXA or siCtrl in the ability to form spheroid. C, CD44 population in MG1, MG2, MG1R, and MG2R GBM cells by flow cytometry analysis. Spheroid formation assay of MG1, MG2, MG1R, and MG2R GBM cells with CD44 or without CD44. D, Quantitative PCR was used to detect the expression of cancer stemness genes in GBM‐MG1, and GBM‐MG2, GBM cells with CD44 or without CD44. E, Quantitative PCR was used to detect the expression of angiogenesis and proliferation genes in GBM‐MG1, and GBM‐MG2, GBM cells with CD44 or without CD44. F, Colony formation assays were performed to compare the radioresistance among GBM‐MG1, and GBM‐MG2, GBM cells with CD44 or without CD44 after radiation (5 Gy). G, Western blotting shows the level of DNA repair protein between GBM‐MG1 and GBM‐MG2 with or without CD44. H, Comet assay and I, Annexin V assay were used to compare the radio‐resistance between GBM‐MG1 and GBM‐MG2 with or without CD44. All data are presented as the mean ± SD, n = 10, *P < .05 by Student's *t* test

A previous study demonstrated that the radioresistance of a glioblastoma presented higher CSC properties.^5^, ^21^, ^22^ Here, we sought to investigate the involvement of GBM‐MG1, GBM‐MG2, GBM‐MG1R, and GBM‐MG2R cells in CSC properties using self‐renewal assays. The spheroid formation assay is a typical assay for examining the ability to self‐renew. Once the cells form a spheroid after several days of culture, then the cells are separated into single cells and allowed to form a new spheroid, which ensures that the stem cell property is indeed maintained by cancer stem cells. The cells were assayed and confirmed with serial generation. Compared to GBM‐MG2 cells, increased sphere numbers were measured in GBM‐MG1 cells, indicating that these cells have self‐renewal potential, whereas GBM‐MG2 cells lost self‐renewal ability. In addition, GBM‐MG1R and GBM‐MG2R cells exhibit higher self‐renewal potential than GBM‐MG1 and GBM‐MG2 cells, respectively (Figure 3A). Then, we performed a spheroid formation assay to identify the gene essential for PTV_95_ tumors in cancer stem cells. We used a small interfering RNA (siRNA) to suppress the candidate genes in the GBM‐MG1 cell line and assayed the tumor spheroid formation ability following siRNA supplementation. The spheroid formation assay revealed that CD44 suppression significantly decreased the number of GBM‐MG1 cell spheroids in vitro (Figure 3B), while siRNAs against DPM2, DAB21, and ANXA slightly decreased the ability to form spheroids. Interesting, we do not observe this effect of CD44 suppression in GBM‐MG2 cell, suggesting that CD44 is not the major factors affecting spheroid formation. To investigate the role of CD44 in GBM with the PTV_95_ program, we analyzed the CD44 population in each cell line. Flow cytometry analysis revealed that GBM‐MG1 cells exhibit higher CD44 expression than GBM‐MG2 cells. Moreover, the radioresistant cell lines GBM‐MG1R and GBM‐MG2R also expressed high levels of CD44 (Figure 3C). To confirm the correlation of CD44 with stemness genes in GBM‐MG1 and GBM‐MG2 cells, we compared the expression of the stemness genes OCT4, NANOG, SOX2, and BMI1 in the presence or absence of CD44. qPCR analysis showed that the expression of stemness genes, including OCT4, NANOG, SOX2, and BMI1, was higher in CD44‐positive GBM‐MG1 cells than in CD44‐negative GBM‐MG1 cells (Figure 3D). We used NESTIN as a negative control because it is broadly expressed in brain tissues. Similarly, stemness gene expression was higher in CD44‐positive GBM‐MG2 cells than in CD44‐negative GBM‐MG2 cells. Considering the results shown in Figure 3C (ie, CD44 expression is higher in GBM‐MG1 cells than in GBM‐MG2 cells), we conclude that the CD44 level plays a crucial role in manipulating cancer stem cell properties and radioresistance. As shown in Figure 2F, H, we found that irradiated GBM cells have high angiogenesis and cell proliferation abilities. Therefore, we used qPCR to examine the mRNA expression of DPM2, DAB2, ANXA1 and Ki67 in GBM‐MG1 or GBM‐MG2 cells in the presence or absence of CD44. The qPCR results showed that the mRNA expression of DPM2, DAB2, ANXA1, and Ki67 expression was upregulated in CD44‐positive GBM‐MG1 and GBM‐MG2 cells, whereas the mRNA expression of DPM2, DAB2, ANXA1 and Ki67 was downregulated in the absence of CD44 (Figure 3E). Consistently, in radiobiological clonogenic assays, the survival abilities of CD44‐positive GBM‐MG1 cells and CD44‐positive GBM‐MG2 were higher than CD44‐negative GBM‐MG1 cells and CD44‐negative GBM‐MG2 cells, respectively, after 5 Gy units (Figure 3F) Moreover, CD44‐positive GBM‐MG1 and GBM‐MG2 cells, with higher radioresistance, exhibited constitutive expression of ATM and RAD51 and CHK2 kinase phosphorylation; however, CD44‐negative GBM‐MG1 and GBM‐MG2 cells, with lower radioresistance, exhibited lower ATM expression and CHK2 kinase phosphorylation (Figure 3G). Furthermore, comet assays performed 24 hours after irradiation revealed that CD44‐positive GBM‐MG1 and GBM‐MG2 cells showed modest DSB accumulation, while CD44‐negative GBM‐MG1 and GBM‐MG2 cells exhibited severe DNA damage (Figure 3H). Annexin V staining revealed that the number of surviving CD44‐positive GBM‐MG1 and GBM‐MG2 cells was significantly higher than that of CD44‐negative GBM‐MG1 and GBM‐MG2 cells (Figure 3I).

Taken together, these data show that CD44 enhances GBM tumorigenesis via radioresistance with a hyperactive DDR, escaping from apoptotic cell death, cancer stem‐like properties, angiogenesis, and cell proliferation.

### 3.4 Upregulation of Ki67 and CD44 expression in clinical samples of recurrent GBM with the PTV_95_ program

**Figure 4:**
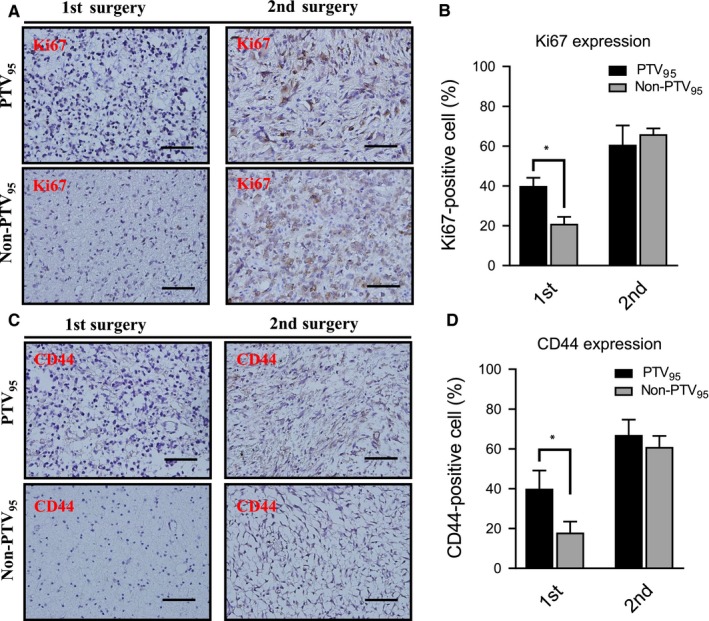
CD44^+^ is abundant in the proliferative cells in GBM patient. A, IHC analysis is used for detecting the level of cell proliferation marker KI67 in the tumor section from PTV_95_ and Non‐PTV_95_ patients. B, The KI67‐positive cells in the sample section were quantified into box plot. C, IHC analysis is used for detecting the level of CD44 in the tumor section from PTV_95_ and Non‐PTV_95_ patient. D, The CD44‐positive cells in the sample section were quantified into box plot. Scale bars, 100 μm. All data are presented as the mean ± SD, n = 10, *P < .01 by Student's *t* test

To confirm the in vitro results, we next investigated the levels of KI67 and CD44 by immunohistochemistry (IHC) staining in samples from two GBM patients. Representative IHC results are shown in Figure 4A,B. These patients received full‐course chemotherapy and IR (PTV_95_ program and Non‐PTV_95_ program, respectively) after their 1st surgery; however, the tumor relapsed, and the patients underwent a second surgery. We observed that the IHC grading of KI67 was higher in recurrent GBM patients than in first diagnosed GBM patients. In addition, the IHC grading of KI67 was similar between the PTV_95_ program and the Non‐PTV_95_ program in recurrent GBM patients. Moreover, the percentage of CD44‐positive cells was dramatically increased in the tumor‐relapse samples compared with the tumor samples from the first surgery (Figure 4C,D). These results suggest that the level of CD44 may be associated with the recurrence of GBM patients. In summary, we found that IR with the PTV_95_ program (a higher radiotherapy dose) resulted in increased radioresistance than IR with the Non‐PTV_95_ program (a lower radiotherapy dose), and the tumor microenvironment (CD44 level) may enhance radioresistance in IR with the Non‐PTV_95_ program.

## 4 DISCUSSION

A previous study found that the general length of survival in GBM patients following diagnosis was 12‐15 months and that less than 3%‐5% of patients survived for more than 5 years.^23^ In our study, we report better survival than in other previous trials. This finding might be due to improvements in modern RT techniques. A reduction in the RT dose to normal tissues outside the PTV is critical, particularly for patients with recurrent malignant glioma who might need to receive RT again. Briere et al^24^ reported that the use of volumetric arc therapy to optimize RT planning in GBM patients provided no distinct advantage and was inferior to conventional RT.

According to our clinical findings, different tumor coverage rates did not improve the prognosis of malignant glioma patients. It appears that even when the PTV coverage can meet the standard therapeutic guidelines, it has no effect on patient survival or disease recurrence. To the best of our knowledge, our study is the first to evaluate whether irradiation coverage affects the clinical prognosis of malignant glioma patients. Even if this result conflicts with current clinical practice due to the lack of a significant difference in OS and PFS, we can perform further studies to evaluate whether it is related to the existence of CSCs in GBM or to the presence of radioinsensitive cells.

An increasing number of studies on CSCs within GBM have highlighted the importance of paracrine signaling networks within the tumor microenvironment on the maintenance and growth of CSCs.^25^ The study of the communication between glioblastoma, CSCs and various cell populations within the brain microenvironment is important not only for determining the biology of GBM but also for predicting the therapeutic response to identify novel targets that could support the prevention of disease recurrence. It was recently determined that the tumor microenvironment is widely influenced by cancer characteristics (i.e, preserving the signals of cell proliferation, activating angiogenesis, escaping from apoptosis, and promoting tumor migration and invasion).^26^

Previous studies have revealed that CD44 is involved in various cellular processes, including cell invasion, proliferation, and apoptosis.^27^, ^28^ It has been reported that CD44 is expressed in many cancers, including brain, colon, breast, prostate, and lung cancers.^29^ In particular, Merzak et al revealed that CD44 expression in GBM promotes the invasion of GBM through cell‐extracellular matrix interactions.^30^ Recent studies have shown that CD44 is a marker of GBM CSCs and that CD44 expression is enriched in GBM CSCs.^31^ In this study, we demonstrated that CD44 is more highly expressed in radioresistant GBM cells and that CD44 plays an important role in stemness, cell proliferation, and angiogenesis.

The short‐lived tumor response after treatment has been associated with the result that RT destroys the bulky GBM but not CSCs, which drive tumor recurrence.^5^ The higher radioresistance of CSCs in GBM compared with nonstem GBM cells is highly correlated with the simultaneous hyperactive DDR^32^ and escape from cell apoptosis.^5^

Previous studies have shown that IR is capable of activating the proliferation of glioma cells that express stem cell markers, such as STAT3, slug, and MSI1, and these results are consistent with the role of CSCs in radioresistance.^5^, ^21^, ^22^ Our previous study revealed that radioresistant CSCs have an activated DNA repair ability, and homologous recombination is a major mechanism underlying the observed radioresistance.^5^, ^21^, ^22^ Moreover, glioblastoma stem‐like cells that repair DSBs are correlated with enhanced activation of the DDR. There are two important DDR signal transducers: (a) CHK2 (whose activation correlates with radioresistance) and (b) ATM (which is responsible for H2AX phosphorylation).^5^, ^33^ In our study, radioresistant GBM‐MG1R2 cells but not GBM‐Par cells were able to rapidly repair DNA damage.

In conclusion, our study showed that IR treatment induced radioresistance and increased the acquisition of stem‐like properties in GBM cells. We suggest that the radioresistance of CSCs is a key feature underlying tumor recurrence because of DNA repair mechanisms. Our results provide insight into the development of new drugs that could reduce the radioresistance that is frequently encountered in current GBM therapies.

## CONFLICTS OF INTEREST

The authors declare that they have no competing interests.

## AUTHORS' CONTRIBUTIONS

JTT, WHL, and JCL made a discussion of study design and regularly followed up the scheduled progress of the experiment. YCC and MHL performed the experimental work and analyzed the data. WHL and JTT provided biological material, patient‐derived cell lines and inform consent. WHL and JCL wrote the manuscript and arranged the figures in sequence. The manuscript was commented by all authors. All authors approved the final version of article.
