# Supplementary material for: CD44‐associated radioresistance of glioblastoma in irradiated brain areas with optimal tumor coverage
Source: Cancer Med. 2019 Nov 19;9(1):350–60. doi: 10.1002/cam4.2714 (PMC6943151; doi:10.1002/cam4.2714)
Supplement: Supplementary file 1 [file CAM4-9-350-s001.docx]

SUPPLEMENTARY INFORMATION

**Supplementary Table 1. The sequences of the primers for quantitative RT-PCR**

| **Gene**  **(Accession No.)** | **Primer Sequence**  **(5’ to 3’)** | **Product size (bp)** | **Tm (°C)** |
| --- | --- | --- | --- |
| OCT4  (NM_002701) | F: TGTGGACCTCAGGTTGGACT  R: CTTCTGCAGGGCTTTCATGT | 207 | 58 |
| NANOG  (NM_024865) | F: TCTTCCTACCACCAGGGATGC  R: CACTGGCAGGAGAATTTGGC | 250 | 59 |
| SOX2  (NM_003106) | F: CGAGTGGAAACTTTTGTCGGA  R: TGTGCAGCGCTCGCAG | 74 | 58 |
| Nestin  (NM_006617) | F: AGGAGGAGTTGGGTTCTG  R: GGAGTGGAGTCTGGAAGG | 112 | 55 |
| BMI1  (NM_005180) | F: AAATGCTGGAGAACTGGAAAG  R: CTGTGGATGAGGAGACTGC | 124 | 57 |
| DPM2  (NM_003863.3) | F: GAAAGCCTGGGGAAGGACTG  R: AGGAGAGGTCAGTCGGTCAG | 115 | 60 |
| DAB2  (NM_001343.4) | F: GGAAGCCAGCAAAGCAGTTG  R: TCTGGTCAACACCCGATTTCA | 89 | 60. |
| ANXA1  (NM_000700.3) | F: AGTTCTTTGCAAGAAGGTAGAGAT  R: CGGGACCACCTTTGGATGAC | 139 | 57 |
| Ki67  (NM_002417.5 | F: CGTCCCAGTGGAAGAGTTGT  R: CGACCCCGCTCCTTTTGATA | 143 | 60 |
| CD44  (NM_000610.3) | F: TTACAGCCTCAGCAGAGCAC  R: TGACCTAAGACGGAGGGAGG | 145 | 60 |
| GAPDH  (NM_002046) | F: CATCATCCCTGCCTCTACTG  R: GCCTGCTTCACCACCTTC | 180 | 58 |

**Bp, base pairs; SOX2, sex determining region Y-box 2; GAPDH, glyceraldehyde 3-phosphate dehydrogenase.Supplementary Table 2. List of proteins tested by antibodies**

| **Protein** | **Assay** | **Antibody** | **Origin** | **Dilution** | **Incubation** |
| --- | --- | --- | --- | --- | --- |
| pATM | WB | rpab | GTX132146, GeneTEX,Inc | 1:2000 | overnight |
| ATM | WB | rpab | GTX111106, GeneTEX,Inc | 1:2000 | overnight |
| pCHK2 | WB | rpab | GTX132204, GeneTEX,Inc | 1:2000 | overnight |
| CHK2 | WB | rpab | #2662, cell Signaling | 1:1000 | overnight |
| RAD51 | WB | rpab | GTX100469, GeneTEX,Inc | 1:1000 | overnight |
| CD44 | IHC, FC | mmab | LS-C758743, LifeSpan | 1:200 | overnight |
| KI67 | IHC | mmab | #9449, Cell Signaling | 1:200 | overnight |
| ß-Actin | WB | mmab | Ab3280, Abcam, Inc | 1:10000 | 2 hrs |

Abbreviations: WB, Western blot; mmab, mouse monoclonal antibody; rmab, rabbit monoclonal antibody; rpab, rabbit polyclonal antibody; IF, immunofluorescence; FC, Flow Cytometry; IHC, Immunohistochemistry.

**Supplementary Table 3. List of siRNA**

| **Name** | **Accession No.** | **Sequence** |
| --- | --- | --- |
| siCtrl | - | AAUUCUCCGAACGUGUCACGU |
| siCD44 | NM_000610.3 | AACGGCUCCUGUUAAAUGGUA |
| siDPM2 | NM_003863.3 | AAGUAUUUCCUGCCCCGAGCC |
| siDAB2 | NM_001343.4 | AAGGUUGGCAUUCCUCAGGAG |
| siMKI67 | NM_002417.5 | AAGACAGCUACAGACUCCUAA |
| siANXA1 | NM_000700.3 | AAGCGAAACAATGCACAGCGT |
